# Relative effectiveness of irish factories in the surveillance of slaughtered cattle for visible lesions of tuberculosis, 2005-2007

**DOI:** 10.1186/2046-0481-65-2

**Published:** 2012-01-30

**Authors:** Francisco Olea-Popelka, Zach Freeman, Paul White, Eamonn Costello, James O'Keeffe, Klaas Frankena, Wayne Martin, Simon More

**Affiliations:** 1 Animal Population Health Institute, Department of Clinical Sciences, College of Veterinary Medicine and Biomedical Sciences, Colorado State University, Fort Collins 80523, USA; 2 Centre of Veterinary Epidemiology and Risk Analysis, University College Dublin, Belfield, Ireland; 3 Department of Agriculture Food and Fisheries, Central Veterinary Research Laboratory, Celbridge, Co. Kildare, Ireland; 4 Department of Agriculture Food and Fisheries, Ireland; 5 Quantitative Veterinary Epidemiology Group, Wageningen Institute of Animal Sciences, PO Box 338, 6700 AH Wageningen, The Netherlands; 6 Department of Population Medicine, Ontario Veterinary College, University of Guelph, Clinical Research Building, Guelph, Ont., Canada N1G 2W1

**Keywords:** Bovine tuberculosis, slaughter surveillance, effectiveness

## Abstract

**Background:**

In Ireland, every animal is examined at slaughter for its fitness for human consumption. The aim of this study was to determine the relative effectiveness of factories in submitting and subsequently in having suspect lesions confirmed as bovine tuberculosis (TB) lesions during the years 2005-2007. This work provides an update from previously published data for years 2003-2004. During 2005-2007 data were available on 4,401,813 cattle from attested herds (*i.e*. herds classified free of bovine TB), from which data for potential confounding factors were available for 3,344,057 slaughtered animals at one of the 37 export-licensed factories.

**Findings:**

From these animals, 8,178 suspect lesions were submitted for laboratory confirmation. Lesions from 5,456 (66.7%) animals tested as positive, and 269 (3.2%) were inconclusive for bovine TB. Logistic regression was used to determine adjusted submission and confirmation risks for each factory while controlling for confounding factors. Factory rankings based on adjusted and crude risks were similar. The average crude submission risk for all the factories was 25 lesions per 10,000 animals slaughtered, ranging from 0 to 52. The crude confirmation risk varied between 30.3% and 91.3%.

**Conclusions:**

Substantial variation in the effectiveness of lesion submission and subsequent confirmation as bovine TB was found among the 37 factories. Compared to previous years (2003-2004), there was an increased bovine TB lesion submission and confirmation risk. Continued monitoring of the effectiveness of slaughter surveillance in Ireland is recommended; emphasis should be placed on efforts to improve bovine TB surveillance in factories with lower rankings.

## Introduction

A programme to eradicate tuberculosis (TB) from cattle was begun by the Irish government in the 1950's [1]. The detection of gross (visible) tuberculous lesions at slaughter has proved to be an essential component of the overall bovine TB surveillance system for the cattle population [1]. With respect to effectiveness of factory surveillance for bovine TB, several studies have been conducted, some based on univariable analysis [2-5] and two using multivariable analysis [6,7]. The multivariable approach is preferable because it helps ensure that measures of surveillance effectiveness are adjusted for factors that can affect the TB status (e.g. age and source) of the animals slaughtered at different factories. This study, using data from 2005-2007, is an update of the evaluation of the effectiveness of factory surveillance in Ireland, first undertaken by Martin *et al*. [6] using data from 2001-2002 and subsequently by Frankena *et al*. [7] using data from 2003-2004. We replicated the analytical methods used by Frankena *et al*. [7] in order to compare the 2005-2007 results with those published for 2003-2004 [7].

## Material and methods

Animal movement, TB tuberculin test, and laboratory results were obtained from the Centre for Veterinary Epidemiology and Risk Analysis (CVERA) at University College Dublin, Ireland. The processing of bovine TB suspected lesions and the interpretation of the results is described by Costello *et al*. [8].

The analytical methodology has been described previously [7]. In summary, a multivariable logistic regression model was developed to calculate the risk of lesion submission for each factory, using animal as the unit of analysis. The model included the following potential confounding factors: the sex, age and origin (homebred or purchased) of each animal, the slaughter season, and, for each animal's herd of origin, the length of the TB clear period for the animal source herd and the District Electoral Division (DED) bovine TB risk. A comparable model was used to analyze the effect of factory on the risk of bovine TB being confirmed in the lesions submitted. The adjusted and crude risks were compared to determine the effect of this adjustment on the estimates of risk and on the ranking of the factories. Bovine TB reactor animals to the single intradermal cervical comparative test were excluded from this study, thus only animals sent for routine slaughter were included in our analysis. All analyses were conducted using SAS v. 9.2 (Statistical Analytical System Institute Inc., Cary, NC).

## Results

Data on a total of 4,401,813 animals were available to calculate the crude risk. The adjusted risk was calculated using data from 3,344,057 of these animals (those with complete data on all confounding factors), from 89,870 attested herds in 2,830 DEDs slaughtered at one of the 37 export-licensed factories. A total of 8,178 suspect lesions were submitted for laboratory confirmation from cattle where there was complete data on all confounding factors. Lesions from 5,456 (66.7%) animals tested as positive, and 269 (3.2%) were inconclusive for bovine TB.

Table 1 presents the number of animals slaughtered in each factory and the estimated crude and adjusted risk of submission per factory. The crude factory submission risk ranged from 0 to 52 per 10,000 animals slaughtered, with an average of 25 (Table 1).

**Table 1:** The crude and adjusted risk of submitting lesions, and their ranking (low to high) for submission, by factory in Ireland during 2005-2007

All animals from the attested herds				Animals from attested herds with complete data for compounding factors				
**Factory**	**Number Slaughtered**	**Crude Risk (%)**	**Crude Rank**	**Number Slaughtered**	**Adjusted Risk (%)**	**Adjusted Rank**	**Crude Risk (%)**	**Crude Rank**

1	29231	0.03	2	24779	0.04	3	0.03	3
2	137742	0.26	20	112028	0.28	16	0.25	21
3	136518	0.20	18	99086	0.24	12	0.18	13
4	49407	0.14	8	33516	0.20	9	0.13	8
5	147355	0.08	3	116494	0.11	5	0.08	4
6	15879	0.43	35	12421	0.52	34	0.48	36
7	25538	0.39	31	20590	0.49	32	0.37	33
8	142768	0.20	15	115803	0.28	17	0.20	17
9	160647	0.16	10	121531	0.21	10	0.17	11
10	114868	0.34	29	80766	0.44	31	0.34	30
11	135827	0.52	37	92378	0.53	35	0.44	35
12	155054	0.27	22	124544	0.38	24	0.26	23
13	171403	0.40	34	129003	0.51	33	0.37	32
14	164152	0.25	19	127606	0.33	21	0.24	19
15	201262	0.32	27	148133	0.40	27	0.31	28
16	125268	0.14	7	79533	0.13	6	0.14	9
17	228150	0.50	36	151398	0.58	37	0.48	37
18	97409	0.36	30	77355	0.44	30	0.32	29
19	8398	0.39	32	5528	0.39	26	0.36	31
20	176361	0.29	25	124360	0.38	23	0.27	24
21	169143	0.20	17	145281	0.30	18	0.20	18
22	46214	0.20	14	37612	0.23	11	0.16	10
23	155831	0.18	12	118032	0.25	13	0.18	12
24	188027	0.18	11	152370	0.26	14	0.19	14
25	167650	0.27	21	129546	0.32	20	0.25	20
26	11089	0.00	1	7512	0.00	2	0.00	1
27	171193	0.12	5	141637	0.16	8	0.10	6
28	168396	0.27	24	130720	0.36	22	0.25	22
29	195648	0.27	23	143011	0.40	28	0.27	25
30	2459	0.08	4	1099	0.10	4	0.09	5
31	166958	0.32	26	128213	0.39	25	0.29	26
32	3224	0.12	6	2448	0.00	1	0.00	1
33	15479	0.15	9	10325	0.14	7	0.11	7
34	260998	0.19	13	193804	0.27	15	0.19	15
35	137159	0.20	16	116786	0.31	19	0.20	16
36	18378	0.34	28	10420	0.41	29	0.29	27
37	100730	0.39	33	78389	0.53	36	0.38	34

**Total**	**4401813**			**3344057**				
**Average**		**0.25**			**0.31**		**0.23**	

After excluding the four factories that submitted suspected lesions from fewer than 10 animals, the adjusted submission risk ranged from 11 to 58 per 10,000 animals slaughtered. The correlation between the adjusted and crude submission risk (based on the data for animals with complete records) was 0.98.

The overall adjusted confirmation risk was 68.5% (Table 2). Among the 33 factories that submitted suspected lesions from at least 10 animals, the confirmation risk varied from 30.2% to 90.1% (Table 2). Crude and adjusted confirmation risks for the animals with complete information about confounding factors were highly correlated (0.99).

**Table 2:** The crude and adjusted risk of confirming lesions, and their ranking (low to high) by factory in Ireland during 2005-2007

All animals from the attested herds				Animals from attested herds with complete data for compounding factors				
**Factory**	**Number Submitted**	**Crude Risk (%)**	**Crude Rank**	**Number Submitted**	**Adjusted Risk (%)**	**Adjusted Rank**	**Crude Risk (%)**	**Crude Rank**

1	8	87.50	35	7	85.71	32	85.71	32
2	365	70.14	20	280	68.64	18	67.50	18
3	277	74.73	28	178	72.88	26	71.91	26
4	69	56.52	6	43	58.11	6	58.14	7
5	113	74.34	26	90	72.68	24	72.22	27
6	69	59.42	8	59	59.27	7	59.32	8
7	100	70.00	19	77	69.53	19	68.83	20
8	288	70.14	21	228	70.43	22	69.30	21
9	259	86.87	34	203	87.80	33	87.68	33
10	396	63.64	10	278	63.81	11	61.87	11
11	710	69.86	18	407	68.12	17	67.32	17
12	422	63.03	9	328	61.96	9	60.98	9
13	687	74.53	27	481	74.83	28	73.18	28
14	417	70.26	22	307	69.95	21	69.38	22
15	652	67.64	15	454	66.09	12	64.32	13
16	170	81.18	31	108	84.13	30	84.26	30
17	1134	73.10	25	721	72.75	25	71.43	25
18	350	64.29	11	245	60.01	8	57.96	6
19	33	30.30	1	20	30.18	1	30.00	1
20	509	55.60	5	331	54.43	4	53.17	4
21	342	68.13	17	292	68.07	16	67.12	16
22	92	77.17	30	59	71.43	23	71.19	24
23	288	65.28	13	207	63.04	10	61.35	10
24	342	81.58	32	284	81.67	29	80.99	29
25	446	71.97	24	322	69.90	20	68.63	19
26	0	-	-	-	-	-	-	-
27	197	85.79	33	148	85.62	31	85.14	31
28	462	67.97	16	332	67.27	14	65.66	14
29	536	66.23	14	388	67.82	15	66.49	15
30	2	50.00	4	1	100.00	35	100.00	35
31	527	70.78	23	368	73.22	27	70.92	23
32	4	75.00	29	-	-	-	-	-
33	23	91.30	36	11	90.91	34	90.91	34
34	505	64.95	12	366	67.08	13	63.93	12
35	277	58.12	7	228	57.05	5	56.14	5
36	63	47.62	3	30	40.06	2	40.00	2
37	396	44.70	2	297	42.42	3	41.08	3

**Total**	**11530**			**8178**				
**Average**		**68.05**			**68.48**		**67.54**	

Correlations between the submission and confirmation risks at each factory were negative and significant (using crude submission and confirmation risks r = -0.44, p = 0.009; using adjusted submission and confirmation risks r = -0.47, p = 0.006).

## Discussion

The observed (crude) factory submission risk ranged from 0 to 52 per 10,000 animals slaughtered (Table 1). There was a five-fold range in submission risk, after excluding factories submitting fewer than 10 animals. The average crude submission risk (25 per 10,000) during 2005-07 was higher than found in previous reports (21.8 per 10,000 [6]; 22 per 10,000, [7]). The variation in crude submission risk among factories (5-fold) was lower than reported previously. There was a 7-fold difference [6] after controlling for year, month and animal type, and a 9-fold difference [7] after controlling for the same confounding factors included in our study). The factory confirmation risk ranged from 30.2% to 90.1%, with an average of 68.5% (this average was slightly higher than the 64.4% previously reported by Frankena *et al*. [7]).

When interpreting these results, care should be taken when drawing direct comparisons between the two study periods. Fewer factories were operational when the later study was conducted (42 for the period 2003-2004 vs. 37 for the period 2005-2007). Furthermore, it is worth noting that the age profile for animals with complete information on confounding factors during the period 2005-2007 differs from those animals in the period 2003-2004. For example, the study population during 2003-2004 [7] included only 0.49% animals older than 8 years old, while in our study, 2.6% of animals were older than 8 years. This may affect the observed (crude) submission and confirmation risk, because age is associated with both lesion submission and confirmation risks [7]. In our study this was confirmed by the increased odds ratios as age of the slaughtered animals increased (data not shown). This difference is because a new EU cattle identification system was introduced in Ireland in 1996 whereby cattle born from that year were registered centrally and also had their birth dates recorded, with older animals retaining their existing old ID (which did not include birth details) until they exited the national herd due to being slaughtered or via natural wastage. Thus, we conducted an additional analysis excluding animals 9-10 years old in which the average submission risk was 24 per 10,000 and the confirmation risk was 67.51%. Additionally we calculated the Pearson correlation coefficients for both the crude submission risk and crude ranking for the 35 factories present in both studies (2003-2004 and 2005-2007). Correlations were 0.76 (p < 0.001) and 0.71 (p < 0.001) respectively. These results indicate that factories with higher crude submission risk (ranking) during 2003-2004 tended to remain with higher crude submission risk (ranking) during the period 2005-2007 and vice versa.

The adjusted risk is the most appropriate descriptive summary of a factory's relative effectiveness (when compared to other factories rankings). The variations in the risk profile of the animals among the factories were substantially less than expected, as shown by the close agreement between the crude and adjusted estimates of the factory risk. Therefore, it can be concluded that the animal- and farm-related factors did not substantially contribute to the variation in factory level submission and confirmation risk that was observed. A similar conclusion was drawn in the earlier study [7]. Collins [9] suggested that the variation in factory surveillance effectiveness may be due to factory-related factors, for example, line speed and light intensity, and/or other factors related to veterinary inspectors, for example their experience, interest, motivation and workload.

There was a negative correlation between the submission risk and the confirmation risk; as the number of submissions increased, the percentage of the lesions that were confirmed as TB decreased. However, we do not think that the proportion of submitted lesions that are confirmed as bovine TB should be a metric for assessing surveillance effectiveness.

The detection of gross bovine TB lesions in cattle at slaughter, coupled with successful trace-back of these animals to the herd of origin, is critical to the detection of infected herds, and for the progress of the national bovine TB control and eradication program in Ireland. Between the years 2005-2007, approximately 30% of new herd breakdowns were identified by means of bovine TB slaughter surveillance [10]. Inadequate inspection of carcasses to find gross (visible) lesions could delay the successful eradication of bovine TB in Ireland and increase the cost of the eradication program. Improved factory surveillance would contribute to national efforts to control bovine TB. The identification of infected herds before the scheduled tuberculin test may help in minimizing the size of major breakdowns in an index herd and the spread of infection from an index herd to contiguous herds.

In conclusion, during the period 2005-2007 an increase in bovine TB lesion submission and confirmation risk was observed when compared to the period 2003-2004. However, substantial variation in both the submission and the confirmation risks of TB lesions among factories remains, indicating that the practices applied in detecting and submitting lesions are not uniform. We suggest that studies be conducted to identify the critical factors (variables) present among factories with "high" and "low" rankings. We also recommend continuing monitoring of the effectiveness of slaughter surveillance in Ireland as part of quality control in the national programme. Emphasis should be placed on efforts to improve bovine TB surveillance in factories identified with lower rankings in this study. Training programmes should be considered that would reduce the variability in submission and confirmation risk measured between individual factories.

## Competing interests

The authors declare that they have no competing interests.

## Authors' contributions

FO: Performed the analysis and interpretation of data. Have been involved in drafting the manuscript and revising it critically for important intellectual content and given final approval of the version to be published. ZF: Contributed to the analysis and interpretation of data. PW: Acquisition of data, have been involved in drafting the manuscript and revising it critically for important intellectual content given final approval of the version to be published. EC: Conducted the work to confirm bovine tuberculosis lesions submitted to the laboratory. JO: Contributions to conception and design, have been involved in drafting the manuscript and revising it critically for important intellectual content and given final approval of the version to be published.

KF: Contributions to conception and design, have been involved in drafting the manuscript or revising it critically for important intellectual content, given final approval of the version to be published. WM: Contributions to conception and design, have been involved in drafting the manuscript or revising it critically for important intellectual content, given final approval of the version to be published. SM: Acquisition of data, have been involved in drafting the manuscript or revising it critically for important intellectual content and given final approval of the version to be published.
